# Protection against severe COVID-19 after second booster dose of adapted bivalent (original/Omicron BA.4-5) mRNA vaccine in persons ≥ 60 years, by time since infection, Italy, 12 September to 11 December 2022

**DOI:** 10.2807/1560-7917.ES.2023.28.8.2300105

**Published:** 2023-02-23

**Authors:** Massimo Fabiani, Alberto Mateo-Urdiales, Chiara Sacco, Emmanouil Alexandros Fotakis, Maria Cristina Rota, Daniele Petrone, Marco Bressi, Martina Del Manso, Andrea Siddu, Giorgio Fedele, Paola Stefanelli, Antonino Bella, Flavia Riccardo, Anna Teresa Palamara, Giovanni Rezza, Silvio Brusaferro, Patrizio Pezzotti, on behalf of the Italian Integrated Surveillance of COVID-19 study group and of the Italian COVID-19 Vaccines Registry group

**Affiliations:** 1 Department of Infectious Diseases, Istituto Superiore di Sanità, Rome, Italy; 2 European Programme on Intervention Epidemiology Training (EPIET), European Centre for Disease Prevention and Control, Stockholm, Sweden; 3 General Directorate of Prevention, Italian Ministry of Health, Rome, Italy; 4 Office of the President, Istituto Superiore di Sanità, Rome, Italy; 5 The members of the group are listed under Acknowledgements

**Keywords:** SARS-CoV-2, COVID-19, bivalent mRNA vaccines, effectiveness, elderly population, Italy

## Abstract

Effectiveness against severe COVID-19 of a second booster dose of the bivalent (original/BA.4–5) mRNA vaccine 7–90 days post-administration, relative to a first booster dose of an mRNA vaccine received ≥ 120 days earlier, was ca 60% both in persons ≥ 60 years never infected and in those infected > 6 months before. Relative effectiveness in those infected 4–6 months earlier indicated no significant additional protection (10%; 95% CI: −44 to 44). A second booster vaccination 6 months after the latest infection may be warranted.

Given the high circulation of severe acute respiratory syndrome coronavirus 2 (SARS-CoV-2) during the year 2022, characterised by predominance of the Omicron variant [1], hybrid immunity conferred by vaccination and infection has become a frequent immunological status in the population, requiring a careful evaluation to refine indications on timing for the administration of a booster dose. Our aim was to evaluate, among persons ≥ 60 years, the effectiveness against severe coronavirus disease (COVID-19) of a second booster dose of the bivalent (original/BA.4–5) mRNA vaccine (BioNtech/Pfizer, Mainz, Germany/New York, United States (US)), relative to a first booster dose of a monovalent vaccine (Comirnaty (BNT162b2 mRNA, BioNtech/Pfizer) or Spikevax (mRNA-1273, Moderna, Cambridge, US) given at least 120 days earlier, according to time elapsed since a prior SARS-CoV-2 infection.

## Study design, data sources, and selection and baseline characteristics of the study participants

We conducted a retrospective cohort study in Italy based on data from the national vaccination registry and the national COVID-19 surveillance system linked through the participants’ individual tax code [2,3]. The study was conducted during predominant circulation of the Omicron BA.5 (Phylogenetic Assignment of Named Global Outbreak lineage (Pango) lineage B.1.1.529.5) subvariant (> 90%) [1]. It started on 12 September 2022 and ended on 11 December 2022. We used data extracted from both sources on 11 January 2023 to account for an additional 28-day period to ascertain possible hospitalisation or death post-infection and 3 days of possible notification delay.

**Figure 1:**
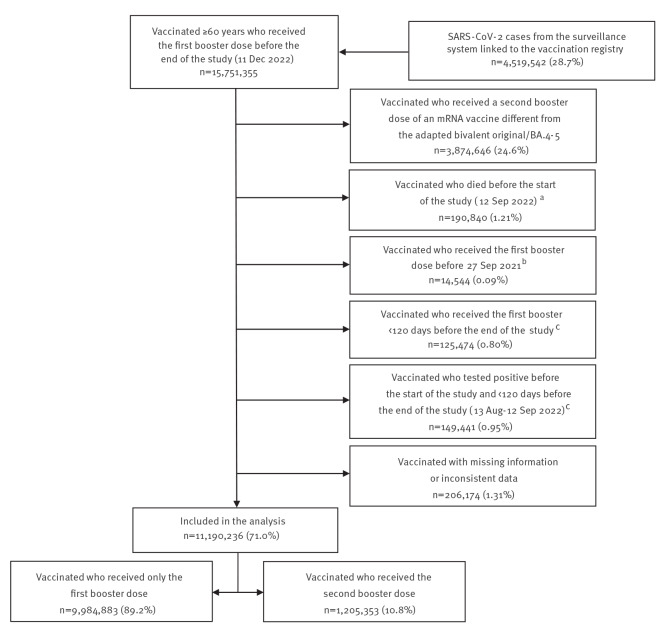
Selection of the COVID-19 vaccine effectiveness study participants, Italy, 14 September 2021–11 December 2022 (n = 15,751,355)

COVID-19: coronavirus disease; SARS-CoV-2: severe acute respiratory syndrome coronavirus 2.

^a^ The expected date of death for individuals who did not receive a diagnosis of SARS-CoV-2 infection during the study period was imputed according to methods described in the Supplementary Methods S1.

^b^ Starting date of the first booster vaccination campaign for persons ≥ 80 years, regardless of frailty status and other high-risk conditions [14].

^c^ Not eligible to receive a second booster dose of vaccine before 11 December 2022 (end date of the study) [10].

We initially selected all individuals aged ≥ 60 years who had received at least the first booster dose of a COVID-19 vaccine before the end of the study. Of these, we excluded 4,561,119 (29.0%) persons not eligible for the study (Figure 1).

The baseline characteristics of the 11,190,236 study participants were quite similar between those who received the first booster dose only and those who received the second booster dose of the bivalent (original/BA.4–5) mRNA vaccine. However, those in the latter group were slightly older, more frequently vaccinated in the northern-central regions of Italy, and reported to have received the first booster or a prior diagnosis of infection earlier than those who received the first booster only (Table 1).

**Table 1:** Baseline characteristics of the COVID-19 vaccine effectiveness study participants, Italy, 12 September–11 December 2022 (n = 11,190,236)

	First booster dose only n = 9,984,883		Bivalent second booster dose (original/BA.4–5) n = 1,205,353		Total n = 11,190,236	
	n	%	n	%	n	%
**Sex**						
Female	5,488,408	55.0	640,702	53.2	6,129,110	54.8
Male	4,496,475	45.0	564,651	46.8	5,061,126	45.2
**Age (years)**						
60–64	2,540,021	25.4	190,399	15.8	2,730,420	24.4
65–69	2,072,467	20.8	234,322	19.4	2,306,789	20.6
70–74	1,849,979	18.5	258,059	21.4	2,108,038	18.8
75–79	1,455,249	14.6	226,286	18.8	1,681,535	15.0
80–84	1,056,862	10.6	154,365	12.8	1,211,227	10.8
85–89	641,339	6.4	91,503	7.6	732,842	6.5
90–94	286,792	2.9	40,231	3.3	327,023	2.9
≥ 95	82,174	0.8	10,188	0.8	92,362	0.8
Median (IQR)	71 (64–78)		73 (67–79)		71 (65–78)	
**Country of birth**						
Italian-born	9,543,362	95.6	1,170,489	97.1	10,713,851	95.7
Foreign-born	441,521	4.4	34,864	2.9	476,385	4.3
**Geographical macro-area**						
North-west	2,405,973	24.1	415,441	34.5	2,821,414	25.2
North-east	1,789,841	17.9	230,849	19.2	2,020,690	18.1
Centre	2,069,489	20.7	314,966	26.1	2,384,455	21.3
South and islands	3,719,580	37.3	244,097	20.3	3,963,677	35.4
**High-risk group^a^**						
No	8,167,077	81.8	1,023,319	84.9	9,190,396	82.1
Yes	1,817,806	18.2	182,034	15.1	1,999,840	17.9
**Weeks since first booster**						
17–26	341,624	3.4	15,447	1.3	357,071	3.2
27–39	6,343,183	63.5	57,279	4.8	6,400,462	57.2
40–66	3,300,076	33.1	1,132,627	94.0	4,432,703	39.6
Median (IQR)	38 (34–40)		47 (44–50)		38 (34–42)	
**Weeks since prior infection**						
No prior infection	7,760,759	77.7	909,194	75.4	8,669,953	77.5
≥ 40	284,454	2.8	74,256	6.2	358,710	3.2
27–39	375,382	3.8	122,342	10.1	497,724	4.4
17–26	1,564,288	15.7	99,561	8.3	1,663,849	14.9
Median number (IQR)^b^	19 (17–31)		30 (24–40)		21 (17–32)	
**First booster vaccine dose**						
Comirnaty (BNT16b2 mRNA)	4,930,409	49.4	683,576	56.7	5,613,985	50.2
Spikevax (mRNA-1273)	5,054,474	50.6	521,777	43.3	5,576,251	49.8

COVID-10: coronavirus disease; IQR: interquartile range; SARS-CoV-2: severe acute respiratory syndrome coronavirus 2.

^a^ Including residents in long-term care facilities and individuals with health risk conditions listed in Supplementary Table S1.

^b^ Among individuals who tested positive for SARS-CoV-2 infection at least 120 days earlier.

**Figure 2:**
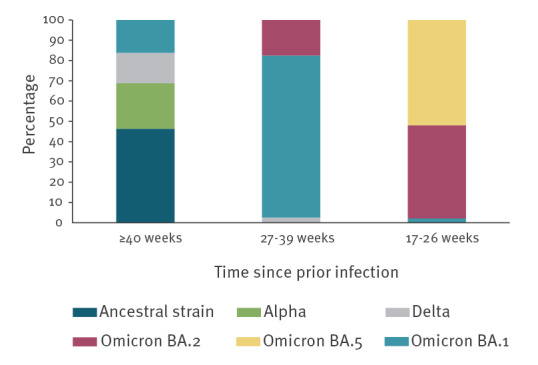
Per cent distribution of SARS-CoV-2 variants associated with prior infections in the study participants, Italy, 21 February 2020–12 August 2022 (n = 2,520,283)

According to estimates of the predominant SARS-CoV-2 variants circulating at different calendar periods [1], most of the participants with a prior infection in the last 17–26 weeks were infected with the Omicron BA.5 (n = 864,102; 52%) or Omicron BA.2 (n = 764,676; 46%) sub-variant, while most of those infected 27–39 weeks earlier were infected with the Omicron BA.1 sub-variant (n = 397,679; 80%). Participants infected ≥ 40 weeks earlier were mostly infected with the ancestral Wuhan strain (n = 166,342; 46%) or the Alpha variant (n = 80,422; 22%) (Figure 2).

SARS-CoV-2: severe acute respiratory syndrome coronavirus 2.

## Relative vaccine effectiveness of a second booster dose of the bivalent original/BA.4–5 mRNA vaccine

We conducted a time-to-event analysis to compare time to infection leading to severe COVID-19 between individuals aged ≥ 60 years who received only the first booster dose of an mRNA vaccine at least 120 days earlier (unexposed group) and those who received a second booster dose of the adapted bivalent (original/BA.4–5) mRNA vaccine (exposed group), according to time elapsed since a prior infection.

According to the case definition based on laboratory criteria, cases of SARS-CoV-2 infections notified to the surveillance system include those who tested positive by PCR or antigenic test [4]. Of these cases, those who were reported to have been hospitalised or died within 4 weeks since infection for COVID-19-related causes were classified as severe cases. According to Italian guidelines, based on indications from the World Health Organization (WHO) [5], a death was considered as related to COVID-19 if it occurred in the presence of a clinical picture suggestive of COVID-19, the absence of a clear cause of death different from COVID-19 (e.g. trauma), and the absence of a complete clinical recovery from the disease. Similarly, the surveillance system foresees and is expected to record only hospitalisations due to SARS-CoV-2 infection and not to other causes.

The follow-up started on 12 September 2022 or later during the study period when an individual became eligible to receive a second booster dose. It ended on the date of testing positive for SARS-CoV-2 infection, date of death or 11 December 2022, whichever came first. The follow-up time was calculated as the number of days elapsed from the starting date to the ending date.

After splitting individual data to account for time-varying vaccination status and calendar week of exposure, we used a multivariable Cox proportional hazard model to estimate the adjusted hazard ratios (HR) of severe COVID-19, according to time elapsed since a prior infection (i.e. no prior infection, ≥ 40 weeks, 27–39 weeks and 17–26 weeks) in persons ≥ 60 years who received the second booster dose of the bivalent original/BA.4–5 mRNA vaccine at least 7 days earlier compared with those who received a first booster dose at least 120 days earlier. Estimates were adjusted for all variables described in Table 1, including age and weeks since the first booster as continuous covariates in the model, and further detailing the geographical area where vaccination took place (i.e. the 19 regions and two autonomous regions of Italy). To account for the calendar week of exposure, we also adjusted the estimates for the weekly regional incidence of SARS-CoV-2 in the general population. The proportional hazard assumption was verified through testing based on Schoenfeld residuals. The relative vaccine effectiveness (rVE) was calculated as (1 − HR) × 100.

Overall, in the time interval 7–90 days after administration of a second booster dose of the bivalent (original/BA.4–5) mRNA vaccine, the rVE against severe COVID-19 was 58.7% (95% confidence interval (CI): 54.6 to 62.5). However, in the same time-interval, rVE differed significantly depending on naturally acquired immunity status (log-likelihood ratio test, p = 0.033) (Table 2).

**Table 2:** Effectiveness against severe COVID-19 of a second booster dose of the bivalent (original/BA.4–5) mRNA vaccine relative to a first booster dose of an mRNA vaccine received ≥ 120 days earlier, by time since prior infection, Italy, 12 September–11 December 2022 (n = 11,190,209)

Time since prior infection	First booster dose since ≥ 120 days (reference)		Bivalent second booster dose (original/BA.4–5)		rVE	
	n events	Rate per 100,000 PD	n events	Rate per 100,000 PD	%	95% CI
**Primary analysis**						
No prior infection	18,594	2.60	413	1.54	59.4	55.1 to 63.3
≥ 40 weeks	433	1.93	17	0.93	61.6	37.5 to 76.3
27–39 weeks	494	1.37	26	0.76	61.7	43.1 to 74.2
17–26 weeks	507	0.52	18	0.73	10.0	−44.0 to 43.8
**Sensitivity analysis^a^**						
No prior infection	13,879	1.94	308	1.15	61.5	56.7 to 65.8
≥ 40 weeks	322	1.43	13	0.71	61.6	33.1 to 78.0
27–39 weeks	353	0.98	17	0.50	65.8	44.3 to 79.0
17–26 weeks	366	0.38	14	0.57	8.5	−56.1 to 46.4

CI: confidence interval; COVID-19: coronavirus disease; PD: person days; rVE: relative vaccine effectiveness.

^a^ Sensitivity analysis based on a more specific definition of severe COVID-19, including death and only admission to a hospital unit usually used for COVID-19 patients (i.e. intensive care unit, semi-intensive care unit, pulmonary unit, infectious and tropical diseases unit or general medicine unit). A total of 5,230 (25.5%) of the 20,502 severe cases identified in the primary analysis were classified as non-severe in the sensitivity analysis.

We did not observe a statistically significant difference in rVE between the group without a prior infection (rVE = 59.4; 95% CI: 55.1 to 63.3) and the groups with a prior infection ≥ 40 weeks (rVE = 61.6, 95% CI: 37.5 to 76.3) or 27–39 weeks earlier (rVE = 61.7; 95% CI: 43.1 to 74.2), while rVE in the group with a more recent prior infection (17–26 weeks before) indicated no additional protection (rVE = 10.0; 95% CI: −44.0 to 43.8) and was significantly lower, considering the Bonferroni correction, compared with the group with a prior infection 27–39 weeks earlier (p = 0.006). The sensitivity analysis based on a more specific definition of severe COVID-19 cases yielded similar results (Table 2).

## Discussion

On 12 September 2022, the European Medicines Agency approved the use of the adapted bivalent (original/Omicron BA.4–5) mRNA vaccine [6], which rapidly became the most used for the booster vaccination campaign in Italy. To date, most European countries recommend a second booster dose to all individuals ≥ 60 years of age and to those ≥ 12 years presenting health-risk conditions who have not recently been infected with SARS-CoV-2 and have not recently received a first booster vaccine dose. However, recommendations regarding the time intervals between previous infection/vaccination and the second booster varies across European countries, with most countries recommending a waiting period between 3 and 6 months [7-9]. In Italy, the current recommendation is 120 days (4 months) [10].

We found that, among persons ≥ 60 years of age in Italy, the rVE of a second booster dose of the adapted bivalent original/BA.4–5 mRNA vaccine in the time-interval 7–90 days post-administration, compared with a first booster dose of a monovalent mRNA vaccine received at least 120 days earlier, was ca 60% in those without a prior infection and in those with a prior infection occurring more than 6 months earlier (≥ 27 weeks). However, we did not observe an additional significant protective effect following the administration of the second booster dose in those infected 4–6 months earlier (17–26 weeks). This suggests that persons aged ≥ 60 years with recently acquired natural immunity might not benefit from a second booster dose received within a period of 6 months post infection, also considering that most of them were previously infected with the Omicron BA.2 and the currently predominant Omicron BA.5 subvariant. Compared with persons recently infected (17–26 weeks), those who had a prior infection ≥ 27 weeks earlier did not acquire natural immunity against the Omicron BA.5 sub-variant and might have benefited more from a second booster vaccination, regardless of time elapsed since prior infection.

Several studies have compared the effectiveness of hybrid immunity against severe COVID-19 due to the Omicron variant [11-13]. However, to our knowledge, there are no studies that investigated the effectiveness of hybrid immunity against severe COVID-19 caused by the Omicron BA.5 subvariant after a second booster dose of an adapted bivalent mRNA vaccine.

Our study is representative of all the population 60 years or older in Italy. It has, however, some limitations. Firstly, although we adjusted the analysis for several covariates, a residual bias due to uncontrolled confounders might remain. Secondly, under-reporting related to self-diagnosis through at-home testing has likely caused an overestimation of people exposed to the risk of severe COVID-19, especially in the first booster group. This could have caused an underestimation of rVE, particularly in those without a notified prior infection. Thirdly, although the Italian surveillance system foresees notification of only those hospitalisations caused by COVID-19, it is possible that cases who incidentally tested positive at admission were misclassified as severe cases. However, the sensitivity analysis restricting the definition of COVID-19-related hospitalisation to the hospital wards where COVID-19 cases are likely to be admitted yielded estimates similar to those from the primary analysis, although a residual bias might remain. An additional sensitivity analysis restricting the definition of severe cases to persons who were admitted to an intensive care unit or died is not presented because of the high level of uncertainty due to the small number of cases meeting this definition. Finally, the study did not include an analysis stratified by age group because the small number of events observed in some strata did not allow robust estimates.

## Conclusions

The results of this study suggest that, for persons ≥ 60 years and with a prior SARS-CoV-2 infection, a second booster dose of the bivalent original/BA.4–5 mRNA vaccine 6 months after the latest infection might be warranted.

## Supplementary Data

196000 Supplement

## References

[1] Italian National Institute of Health (ISS). EpiCentro. Monitoraggio delle varianti del virus SARS-CoV-2 di interesse in sanità pubblica in Italia – Indagini rapide. [Monitoring of the SARS-CoV-2 variants of interest for public health in Italy – rapid investigations]. Italian. Rome: ISS. [Accessed: 1 Feb 2023]. Available from: https://www.epicentro.iss.it/coronavirus/sars-cov-2-monitoraggio-varianti-indagini-rapide

[2] Italian Government, Presidency of the Council of Ministers. Report vaccini anti COVID19. [COVID-19 vaccination report]. Rome: Italian Government; 2020. Italian. Available from: https://www.governo.it/it/dipartimenti/commissario-straordinario-lemergenza-covid-19/15974

[3] Italian National Institute of Health (ISS). COVID-19 integrated surveillance: key national data. Rome: ISS. [Accessed: 1 Feb 2023]. Available from: https://www.epicentro.iss.it/en/coronavirus/sars-cov-2-integrated-surveillance-data

[4] European Centre for Disease Prevention and Control (ECDC). Case definition for coronavirus disease 2019 (COVID-19), as of 3 December 2020. Stockholm: ECDC. [Accessed: 1 Feb 2023]. Available from: https://www.ecdc.europa.eu/en/covid-19/surveillance/case-definition

[5] World Health Organization (WHO). International guidelines for certification and classification (coding) of COVID-19 as cause of death. Geneva: WHO; 2020. Available from: https://www.who.int/publications/m/item/international-guidelines-for-certification-and-classification-(coding)-of-covid-19-as-cause-of-death

[6] European Medicines Agency (EMA). Adapted vaccine targeting BA.4 and BA.5 Omicron variants and original SARS-CoV-2 recommended for approval. Amsterdam: EMA; 2022. Available from: https://www.aifa.gov.it/documents/20142/1621464/2022.09.13_com-EMA_raccomandazione_vaccino_adattato_Comirnaty_B4-5_EN.pdf

[7] Robert Koch Institute (RKI). STIKO: 24. Aktualisierung der COVID-19-Impfempfehlung. [STIKO: 24th update of the COVID-19 vaccination recmmendations]. Epid Bull. 2022;50. German. Available from: https://www.rki.de/DE/Content/Infekt/EpidBull/Archiv/2022/Ausgaben/50_22.pdf?__blob=publicationFile

[8] Vaccination Info Service. Le vaccin contre La Covid-19. [The COVID-19 vaccine]. Paris: Santé publique France. [Accessed: 1 Feb 2023]. French. Available from: https://vaccination-info-service.fr/Les-maladies-et-leurs-vaccins/Covid-19

[9] Sistema Nacional de Salud. Consejo Interterritorial. Actualización de las recomendaciones de vacunación frente a COVID-19 para el otoño-invierno en España. [Update on the recommendations of vaccination against COVID-19 for autumn-winter in Spain]. Madrid: Ministerio de Sanidad. [Accessed: 1 Feb 2023]. Spanish. Available from: https://www.sanidad.gob.es/profesionales/saludPublica/prevPromocion/vacunaciones/covid19/docs/Recomendaciones_vacunacion_Otono_Invierno_Covid.pdf

[10] Italian Medicines Agency (AIFA). Modifica della determina n. DG/153/2022 dell'11 aprile 2022 di inserimento dell'indicazione «seconda dose booster» dei medicinali «Comirnaty» e «Spikevax» nell'elenco dei medicinali ai sensi della legge 23 dicembre 1996, n. 648. (Determina n. DG/301/2022). (22A04093) (GU Serie Generale n.161 del 12-07-2022). [Amendment of resolution no. DG/153/2022 of 11 April 2022 on the inclusion of the indication ”second booster dose” of the medicines ”Comirnaty” and ”Spikevax” in the list of medicines pursuant to the law of 23 December 1996, n. 648. (Decision no. DG/301/2022). (22A04093) (GU General Series n.161 of 12-07-2022)]. Official Gazette of the Italian Republic. 2022;163(161):14. Italian. Available from: https://www.gazzettaufficiale.it/eli/gu/2022/07/12/161/sg/pdf

[11] Bobrovitz N Ware H Ma X Li Z Hosseini R Cao C Protective effectiveness of previous SARS-CoV-2 infection and hybrid immunity against the omicron variant and severe disease: a systematic review and meta-regression. Lancet Infect Dis. 2023;S1473-3099(22)00801-5. 10.1016/S1473-3099(22)00801-5 36681084 PMC10014083

[12] Malato J Ribeiro RM Fernandes E Leite PP Casaca P Antunes C Stability of hybrid versus vaccine immunity against BA.5 infection over 8 months. Lancet Infect Dis. 2023;23(2):148-50. 10.1016/S1473-3099(22)00833-7 36620968 PMC9815828

[13] Hansen CH Friis NU Bager P Stegger M Fonager J Fomsgaard A Risk of reinfection, vaccine protection, and severity of infection with the BA.5 omicron subvariant: a nation-wide population-based study in Denmark. Lancet Infect Dis. 2023;23(2):167-76. 10.1016/S1473-3099(22)00595-3 36270311 PMC9578720

[14] Italian Ministry of Health. Avvio della somministrazione di dosi “booster” nell’ambito della campagna di vaccinazione anti SARS-CoV-2/COVID-19. [Start of the administration of booster doses as part of the anti-SARS-CoV-2/COVID-19 vaccination campaign]. Rome: Ministero della Salute; 2021. Italian. Available from: https://www.trovanorme.salute.gov.it/norme/renderNormsanPdf?anno=2021&codLeg=82953&parte=1%20&serie=null

