# Supplementary material for: Protection against severe COVID-19 after second booster dose of adapted bivalent (original/Omicron BA.4-5) mRNA vaccine in persons ≥ 60 years, by time since infection, Italy, 12 September to 11 December 2022
Source: Euro Surveill. 2023 Feb 23;28(8):2300105. doi: 10.2807/1560-7917.ES.2023.28.8.2300105 (PMC9951255; doi:10.2807/1560-7917.ES.2023.28.8.2300105)
Supplement: Supplement [file 23-00105_FABIANI_Supplement.pdf]

This supplementary material is hosted by Eurosurveillance as supporting information alongside the article *Protection against severe COVID-19 following a 2nd booster dose of the adapted bivalent (original/Omicron BA.4-5) mRNA vaccine in persons aged ≥60 years according to time from prior infection (Italy, 12 September 2022 to 11 December 2022)*, on behalf of the authors, who remain responsible for the accuracy and appropriateness of the content. The same standards for ethics, copyright, attributions and permissions as for the article apply. Supplements are not edited by Eurosurveillance and the journal is not responsible for the maintenance of any links or email addresses provided therein.

SUPPLEMENTARY MATERIAL

TABLE OF CONTENTS

	Page
**Supplementary Method S1**. Imputation of the expected date of death for vaccinated individuals who did not receive a diagnosis of SARS-CoV-2 during the study period (12 September 2022 to 11 December 2022)	3
**Supplementary Table S1**. High risk conditions recorded into the national vaccination registry	4

Supplementary Method S1.

Imputation of the expected date of death for vaccinated individuals who did not receive a diagnosis of SARS-CoV-2 during the study period (12 September 2022 to 11 December 2022).

We used the life tables by region, age, and sex for the year 2019, published by the Italian Institute of Statistics (http://dati.istat.it/Index.aspx?DataSetCode=DCIS_MORTALITA1), reporting the yearly probability of death $\mathbf{q_{x,y,z}}$ (per 1000)**,** where x,y, and z indicate the region, age and sex, respectively.

Based on a uniform distribution, we randomly extracted and assigned a number in the interval 0-1 to every individual who did not receive a diagnosis of SARS-CoV-2 infection after the start of the study (12 September 2022), assuming it as the cumulative probability of death **S(t)**.

Based on the survival exponential function

$$\mathbf{S(t) = \exp(-q_{x,y,z} * t)},$$

we then calculated for each of these individuals the expected number of survival days after the last known surviving date (i.e, the latest between the date of last vaccine dose administration or 29 days after the last infection date, considering 28 days the maximum length of follow-up post-infection to ascertain a possible death in the surveillance system) as:

$$\mathbf{t = -\log[S(t)]/[q_{x,y,z}/(365*1000)]}.$$

Among the 15,751,355 vaccinated individuals ≥60 years of age who received at least the first booster vaccine dose by 10 December 2022, we imputed a total of 241,175 (1.53%) deaths estimated to be occurred before the end of follow-up (11 December 2022) for causes unrelated to COVID-19.

Supplementary Table S1. High-risk conditions recorded into the national vaccination registry.

Description
Residents in long-term care facilities
Cystic fibrosis
Defects of the complement system. Other specified disorders involving the immune mechanism; Deficiency or dysfunction of a single component (C1-C9)
Human immunodeficiency virus [HIV] disease, Human immunodeficiency virus, type 2 [HIV-2], Asymptomatic human immunodeficiency virus [HIV] infection status
Disorders involving the immune mechanism
Chronic Alcohol Misuse
Functional or anatomic asplenia
COPD
Chemotherapy or Radiotherapy
Coagulopathies
Diabetes Mellitus and other endocrinopathies
Patients in hemodialysis or with chronic kidney diseases expected to start dialysis
Hemoglobinopathy such as sickle cell anemia or thalassemia
Chronic Liver Disease
Cochlear implant
Chronic Kidney Disease
Chronic eczema or psoriasis
Diseases associated with a high risk of aspiration pneumonia
Chronic Cardiovascular Disease
Chronic Respiratory Disease
Motor neuron diseases
Chronic inflammatory diseases and malabsorption syndromes
Blood cancers (leukemia, lymphoma and myeloma)
Solid tumors
Obesity
Bone marrow transplant
Drug Misuse
Solid organ transplant
Patients with CSF leak from trauma or intervention
Patients in immunosuppressive treatment
Metabolic diseases
Hematopoietic diseases
Pathologies that require important surgical interventions
Neurological diseases
Cerebrovascular diseases
Down Syndrome
Disabilities (physical, sensorial, learning or psychic)
